# Study of Cytotoxicity of Pyrrolo[3,4-*d*]isoxazoline and Pyrrolo[2,1-*a*]isoquinoline Derivatives Against Tumor Cell Lines

**DOI:** 10.3390/ijms27146231

**Published:** 2026-07-13

**Authors:** Andrew S. Drachuk, Sergey S. Mkrtchan, Stanislav V. Shmakov, Sergey Yu. Vyazmin, Kristina A. Kim, Alexander V. Stepakov, Vitali M. Boitsov

**Affiliations:** 1 Laboratory of Nanobiotechnologies, Alferov University, ul. Khlopina 8/3, 194021 St. Petersburg, Russia; 2 Department of Organic Chemistry, Saint Petersburg State Institute of Technology (Technical University), Moskovsky prospect 26, 190013 St. Petersburg, Russia

**Keywords:** pyrrolo[3,4-*d*]isoxazolines, pyrrolo[2,1-*a*]isoquinolines, antiproliferative activity, morphological changes (cytoskeleton), cell motility, apoptotic activity, tumor cell lines

## Abstract

Antiproliferative activity of pyrrolo[3,4-*d*]isoxazolines and pyrrolo[2,1-*a*]isoquinolines derived from them was studied against human erythroleukemia (K562), cervical carcinoma (HeLa), and melanoma (Sk-mel-2) cell lines in vitro by MTS assays followed by study of their effect on actin cytoskeleton and cell motility by confocal microscopy, and apoptotic activity by flow cytometry. Most effective among the screened compounds were bicyclic hydroxylactams **6**–**9** with a pyrrolo[3,4-*d*]isoxazoline structure; they showed IC_50_ values ranging from 12 to 36 μg/mL for all tested cancer cell lines with selectivity indexes up to 12 (as compared to the embryonic kidney HEK293T cell line). Loss of stress fibers with diffuse redistribution of granular actin throughout the cytoplasm in up to 25% of treated cells and a decrease in filopodia-like protrusions up to 69% were observed by confocal microscopy during an actin cytoskeleton study. Such cytoskeletal changes and the proposed altered cell motility were confirmed by scratch-test (revealed a three-fold decrease in cell motility).

## 1. Introduction

**Figure 1:**
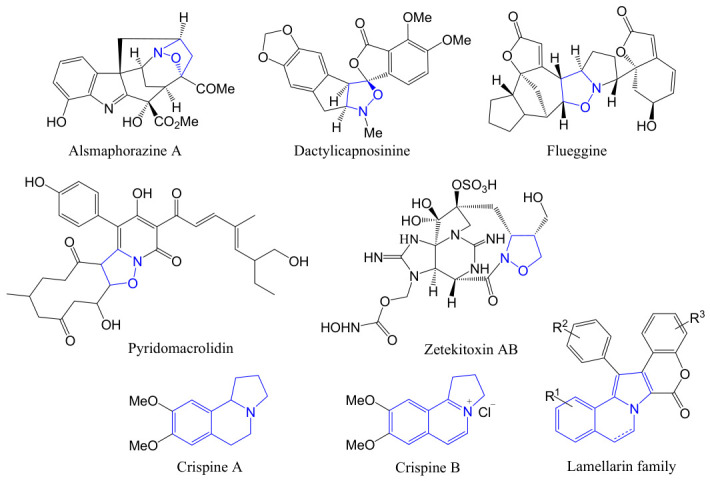
Pyrrolo[2,1-*a*]isoquinoline and isoxazolidine moieties in natural products.

Cancer is one of the most frequently occurring and life-threatening health conditions around the world. Natural compounds obtained from microorganisms, animals, and plants are a major source of inspiration for developing anticancer drugs, as over 50% of all approved anticancer therapies—and many agents in clinical trials—are either natural products or chemically modified derivatives of them [1,2,3]. Among various heterocyclic systems, the pyrrolo[2,1-*a*]isoquinoline framework which combines two privileged moieties occurs frequently in a large number of bioactive natural products (crispines A/B, lamellarin family alkaloids) and pharmaceutically important molecules [4,5,6,7], with shown anticancer activity among other benefits [8,9,10]. 1,3-Dipolar cycloaddition and multicomponent reactions are generally used to construct pyrrole moiety of the pyrrolo[2,1-*a*]isoquinolines [11,12,13], while creation of the isoquinoline moiety was often achieved by C–H activation reactions [14,15,16]. Fusion of the other two privileged moieties forms the favored pyrrolo [3,4-d] isoxazoline scaffold in medicinal chemistry, which is of interest due to its wide range of bioactivities [17,18,19]. Isoxazole, isoxazoline, and isoxazolidine derivatives—(un)saturated five-membered rings containing both nitrogen and oxygen atoms—are regarded as key core scaffolds in organic and medicinal chemistry due to their presence in many biologically important compounds exhibiting varied activities (Figure 1) [20,21,22,23,24]. Such previous studies revealed that compounds containing these moieties possess anti-inflammatory [25,26], antimicrobial [27,28], antiviral [29,30], and anticancer [31,32,33] activities. 1,3-Dipolar cycloaddition reactions are typically utilized to construct five-membered ring systems [34,35,36,37,38,39]. Cycloaddition of nitrones or nitrile oxides as 1,3-dipoles and a variety of alkenes as dipolarophiles is considered one of the important protocols for the synthesis of isoxazole, isoxazoline, and isoxazolidine ring systems [40,41,42,43,44,45,46].

Here, in continuation of our earlier works, we report an in vitro antiproliferative activity study of some pyrrolo[3,4-*d*]isoxazolines and derived from them pyrrolo[2,1-*a*]isoquinolines against selected tumor cell lines (human erythroleukemia (K562), cervical carcinoma (HeLa), and melanoma (Sk-mel-2)) as well as their effect on actin cytoskeleton, cell motility, and apoptotic activity.

## 2. Results and Discussion

### 2.1. Chemistry

**Figure 2:**
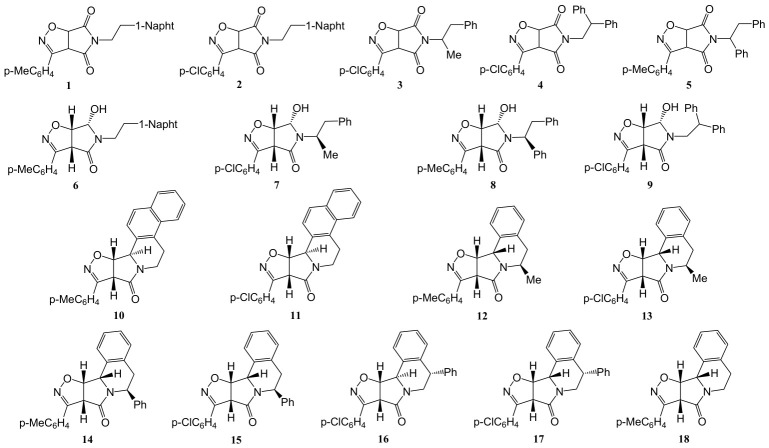
Structures of pyrrolo[3,4-*d*]isoxazolines **1**–**9** and pyrrolo[2,1-*a*]isoquinolines **10**–**18**.

The desired pyrrolo[3,4-*d*]isoxazolines were synthesized by our previously developed methodology via 1,3-dipolar cycloaddition of substituted maleimides to nitrile oxides generated in situ from the corresponding hydroximoyl chlorides in the presence of trimethylamine. Subsequent reduction of the imide function with NaBH_4_ gave hydroxylactams, which were further converted to corresponding pyrrolo[2,1-*a*]isoquinolines via BF_3_*OEt_2_-catalyzed intramolecular diastereoselective Friedel-Crafts-type cyclization [47,48,49]. The structures used in this study, racemic pyrrolo[3,4-*d*]isoxazolines **1**–**9** and pyrrolo[2,1-*a*]isoquinolines **10**–**18**, are shown in Figure 2.

In silico ADMET properties prediction Table 1 and Table 2 present the results of the analysis of physicochemical profiles and pharmacokinetic properties of the studied products. Since pharmacokinetic parameters that involve absorption, distribution, metabolism, excretion and toxicity (ADMET) are mostly dependent on therapeutic usage, pharmacokinetic evaluation is thought of as the initial screening for proposed drugs [50,51,52]. The logP and surface area ranges, as well as the quantity of hydrogen acceptors and donors, are all met by the studied products, which have a molecular weight of less than 500 g/mol. This means observing the Lipinski rule and the oral drugability of the products. In order to evaluate the pharmacokinetic characteristics, ADMET screening was also carried out, and it revealed acceptable ranges for human intestinal absorption and carcinogenicity. The outcomes for cycloadducts for descriptors such as in vivo blood–brain barrier penetration, Cytochrome P450 2D6 inhibition, mutagenicity according to the Ames test, and human ether-a-go-go related gene channel inhibition cover a wide range. The values of the studied products’ bioactivity are displayed in Table 2. Overall, pharmacokinetic analysis indicates that the studied products have the characteristics necessary for medication absorption.

### 2.2. Biology

**Figure 3:**
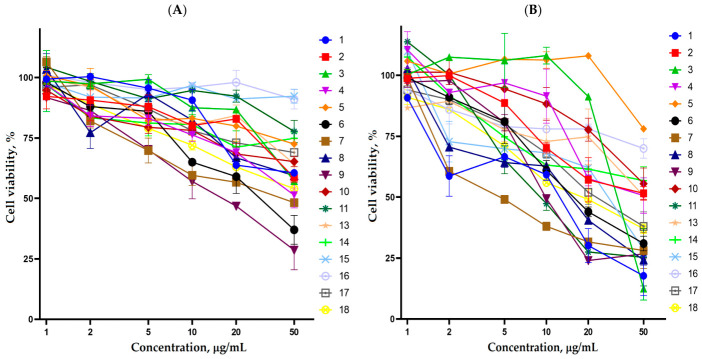
Cytotoxicity of racemic pyrrolo[3,4-*d*]isoxazolines **1**–**9** and pyrrolo[2,1-*a*]isoquinolines **10**–**18** against K562 cell line for 24 (**A**) and 72 (**B**) h.

**Figure 4:**
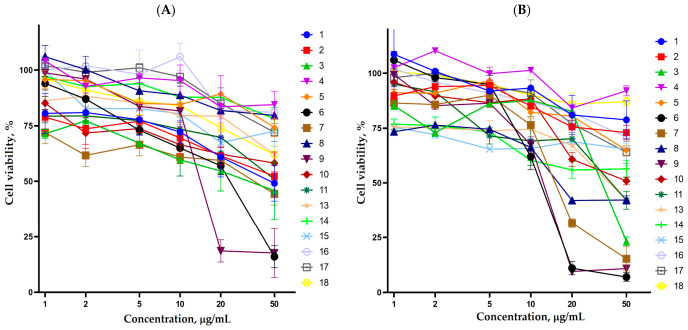
Cytotoxicity of racemic pyrrolo[3,4-*d*]isoxazolines **1**–**9** and pyrrolo[2,1-*a*]isoquinolines **10**–**18** against HeLa cell line for 24 (**A**) and 72 (**B**) h.

**Figure 5:**
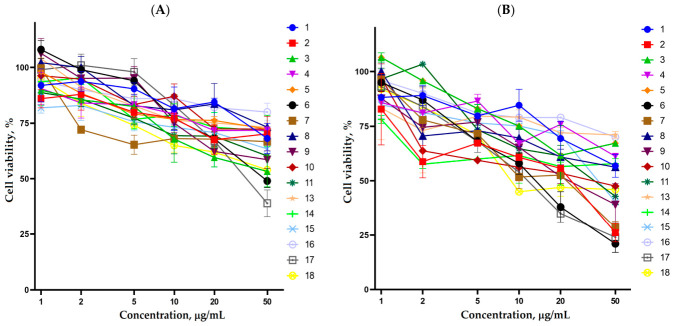
Cytotoxicity of racemic pyrrolo[3,4-*d*]isoxazolines **1**–**9** and pyrrolo[2,1-*a*]isoquinolines **10**–**18** against Sk-mel-2 cell line for 24 (**A**) and 72 (**B**) h.

Cancer cell lines serve as useful in vitro models extensively employed in cancer research and drug discovery because they supply an essentially unlimited amount of biological material for experiments [53,54,55]. In this study, an in vitro MTS assay was used to evaluate the antiproliferative effects of pyrrolo[3,4-*d*]isoxazolines **1**–**9** and pyrrolo[2,1-*a*]isoquinolines **10**–**18** on human erythroleukemia (K562), cervical carcinoma (HeLa), and melanoma (SK-MEL-2) cell lines [56,57]. It was found that the studied derivatives **1**–**18** significantly reduced cell proliferation in a time- and concentration-dependent manner. The results of these investigations are presented in Figure 3, Figure 4 and Figure 5. IC_50_ values of the most active derivatives against the tested cell lines for 72 h are presented in Table 3.

It is obvious from the obtained data that bicyclic hydroxylactams **6**–**9** were more effective among the screened compounds with IC_50_ values ranging from 12 to 36 mg/mL and selectivity indexes up to 12. In general, non-reduced bicyclic isoxazolines **1**–**5** and prepared by cyclization of hydroxylactams isoxazolopyrroloisoquinolines **10**–**18** showed lower activity. At the same time, isoxazolopyrroloisoquinolines with a 4-chlorophenyl-substituted isoxazoline moiety (**11**, **13**, **15**) showed better results as compared to 4-tolyl-substituted derivatives (**10**, **12**, **14**).

For compounds that demonstrated stronger antiproliferative activity, the selectivity indexes (SI) were determined compared to human embryonic kidney cells HEK293T (Table 4).

Tested compounds showed comparable antiproliferative effect (IC_50_ range 12–37 μg/mL, that equal to 28–80 μM) to related classes of compounds—pyrrolo[2,1-*a*]isoquinolines and pyrrolo[3,4-*d*]isoxazolines. Indeed, Crispine B showed antiproliferative activity with an IC_50_ value of about 25 μg/mL against the HeLa cell line [8]; synthetic derivatives of crispine family alkaloids showed activity to sub-micromolar concentration [58,59]. Structurally more complicated lamellarins and their synthetic analogues showed activity in the low micromolar range generally (4–50 μM), while for a few of them (Lamellarins D, N, and synthetic Dehydrolam. J), activity to sub-nanomolar concentration (0.1 nM) was reported [60]. Cytotoxic activity in the 5–90 μM range was reported for pyrrolo[3,4-d]isoxazolines [17].

Compounds that demonstrated stronger antiproliferative activity were selected for additional studies of cytoskeletal morphology, cell migration, and apoptotic activity.

Actine Cytoskeleton Changes

The actin cytoskeleton is crucial for preserving cell morphology, resisting mechanical deformation, and driving key processes such as cell division, endocytosis, and migration [61,62,63]. Filamentous actin (F-actin) is a major structural component of the eukaryotic cytoskeleton, abundant in both muscle and non-muscle cells, where it provides mechanical support and enables cellular movement. Within cells, F-actin is highly dynamic, polymerizing and depolymerizing in response to signaling cues, which is vital for cytoskeletal remodeling and cellular adaptability. Dysregulation of actin dynamics or mutations in actin-associated proteins can contribute to pathological states, such as cancer [64,65,66]. For instance, during metastasis, cancer cells harness F-actin dynamics to increase motility. Thus, F-actin is not only a fundamental structural element but also a key target for investigating disease mechanisms and identifying potential therapeutic intervention points [67,68]. Visualization of these dynamic structures commonly uses actin staining, typically with phalloidin [69,70]. Phalloidin binds F-actin and strongly inhibits depolymerization, reducing ATP-dependent filament disassembly, and thereby stabilizing the existing actin architecture. So, fluorescently labeled phalloidin is widely used to image this “frozen” cytoskeletal state [71].

The structure of the actin cytoskeleton of HeLa and Sk-mel-2 cells was assessed after cultivation with pyrrolo[3,4-*d*]isoxazolines and pyrrolo[2,1-*a*]isoquinolines by the presence of filopodia-like protrusions and the availability of stress fibers.

**Figure 6:**
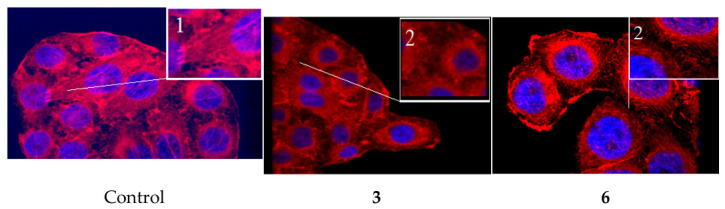

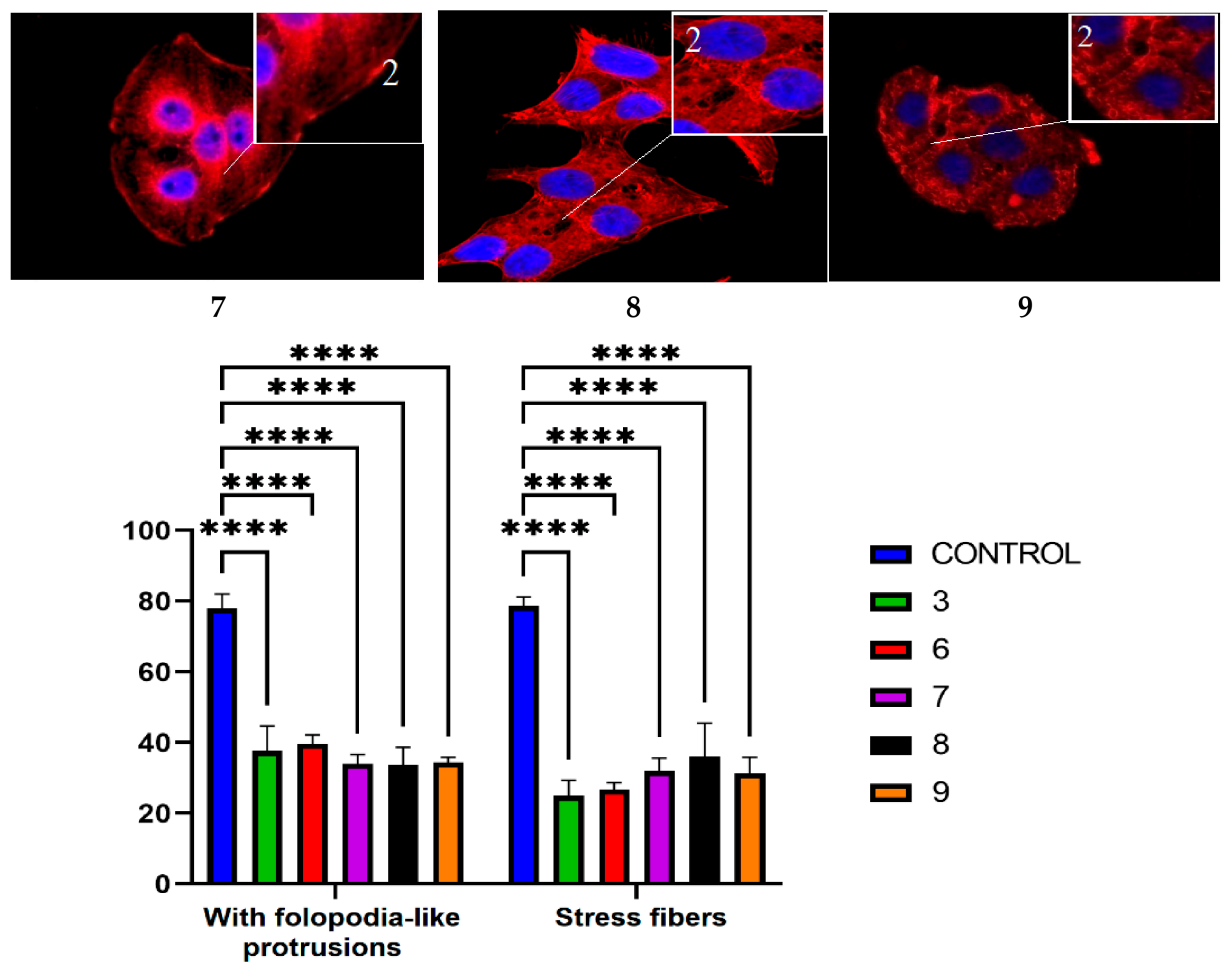
Microscopic images of treated cells and state of actin cytoskeleton of HeLa cells after cultivation with compounds **3, 6**–**9** (10 μg/mL). Inserts: 1—stress fibers; 2—disassembled stress fibers. *p* value < 0.0001 (****).

**Figure 7:**
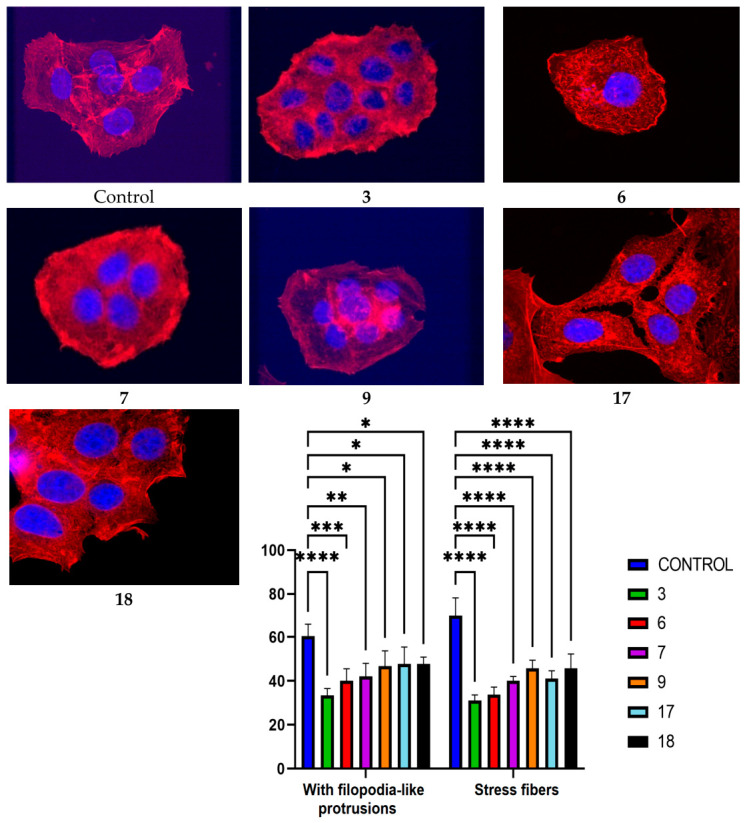
Microscopic images of treated cells and state of actin cytoskeleton of Sk-mel-2 cells after cultivation with compounds **3**, **6**, **7**, **9**, **17**, **18** (10 μg/mL). *p* value < 0.05 (*), 0.01 (**), 0.001 (***), 0.0001 (****).

Using confocal microscopy, we observed that treatment of HeLa cells with the tested derivatives **3** and **6**–**9** induced pronounced alterations in the actin cytoskeleton. These changes included loss of stress fibers, with granular actin redistributed diffusely throughout the cytoplasm in up to 25% of treated cells, as well as a marked decrease in filopodia-like protrusions, which was reduced by as much as 69% after incubation. Such cytoskeletal changes may indicate altered cell motility, which could in turn suggest a decreased metastatic capacity of the tumor cells. Meanwhile, the experimental effects did not induce nuclear fragmentation, suggesting a lack of pro-apoptotic activity. A comparable effect on the actin cytoskeleton was also detected in Sk-Mel-2 cells following exposure to the studied compounds. The actin cytoskeleton structure, along with charts illustrating the percentages of cells with filopodia-like deformations and disrupted stress fibers, is summarized in Figure 6 and Figure 7.

Inhibition of Cell Motility Evaluated by Scratch-Test

Cell motility is one of the most fundamental and evolutionarily conserved cellular behaviors and plays a key role in invasion and metastasis. Cancer metastasis, defined as the spread of tumor cells from the primary site to distant organs, remains a major clinical challenge in cancer diagnosis and treatment. A key difficulty in studying metastatic progression is that this process cannot be directly observed or manipulated in real time. Inhibition of cell migration is often accompanied by pronounced morphological changes and reorganization of the actin cytoskeleton. The scratch assay is a simple model used to assess the effects of different factors on cell motility and metastatic behavior [72,73].

To assess the potential ability of pyrrolo[3,4-*d*]isoxazolines **3** and **6**–**9** to inhibit metastasis associated with cell motility, a Scratch-test was performed on human cervical carcinoma and melanoma cell lines.

**Figure 8:**
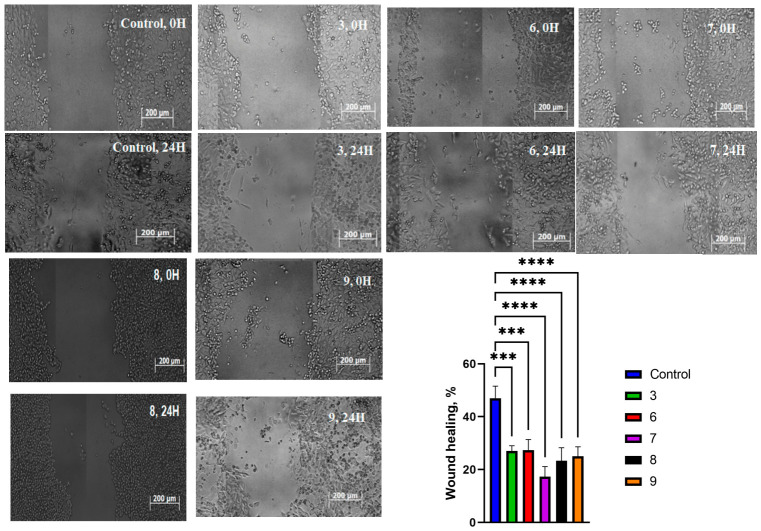
Microscopic images of the HeLa cell wound area in the scratch assay and wound area (%) in the scratch assay after 24 h incubation with pyrrolo[3,4-*d*]isoxazoles **3**, **6**–**9**. *p* value < 0.001 (***), 0.0001 (****).

**Figure 9:**
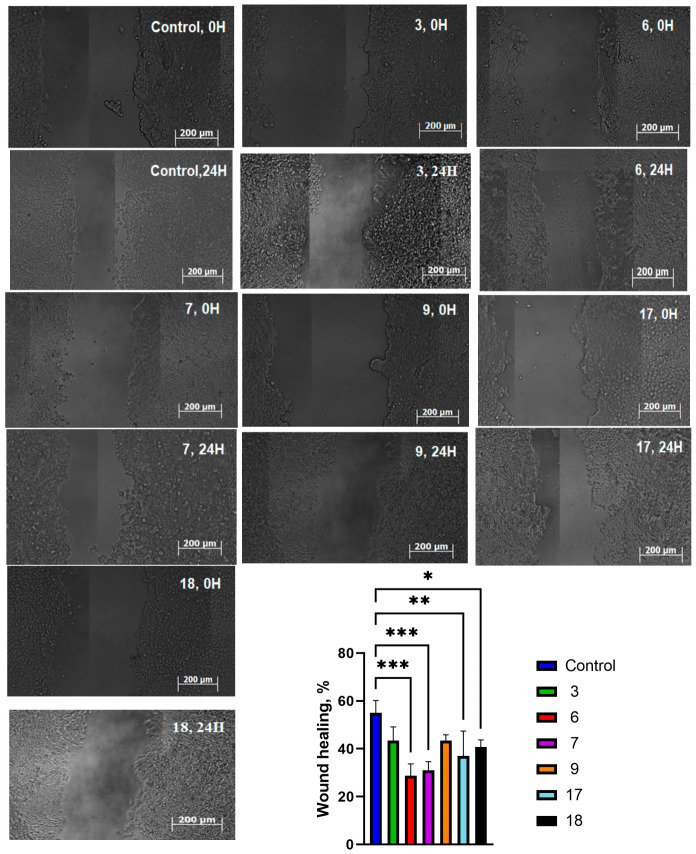
Microscopic images of Sk-mel-2 cells wound area in the scratch assay and wound area (%) in the scratch assay after 24 h incubation with pyrrolo[3,4-*d*]isoxazoles **3**, **6, 7**, **9**, **17**, **18**. *p* value < 0.05 (*), 0.01 (**), 0.001 (***).

Different fields were analyzed by bright field; for fast and non-toxic cell visualization, each scratch area was photographed at 0 and 24 h. The results are shown in Figure 8 and Figure 9. Nontreated HeLa cells filled the scratched strip at 45 ± 5%, while under treatment with pyrrolo[3,4-*d*]isoxazolines **3** and **6**–**9,** cells filled 25 ± 4, 25 ± 5, 15 ± 5, 21.0 ± 5, and 23 ± 4% of the scratched strip, respectively. A comparable effect was also observed for Sk-Mel-2 cells following exposure to the studied compounds: nontreated cells filled the scratched strip at 55 ± 5%, while under treatment with pyrrolo[3,4-*d*]isoxazolines **3** and **6**–**9,** cells filled 42 ± 5, 29 ± 6, 32 ± 4, 41.0 ± 3, 38 ± 7% and 40 ± 2% of the scratched strip, respectively.

Therefore, cells treated with HeLa and Sk-mel-2 lose their ability to move and do not fill the scratched strip. The presented results indicate that the tested compounds can block the cellular movement of tumor cells.

It can be assumed that the tested compounds exhibit both antiproliferative and antimigratory effects. We demonstrated that treatment with these compounds induces reorganization of the actin cytoskeleton, which may reflect decreased cell motility. Together with the scratch assay results, this suggests that tested compounds impair cell migration. Additionally, compounds showing stronger antiproliferative activity tended to display greater antimigration effects [74].

Detection of Apoptosis

**Figure 10:**
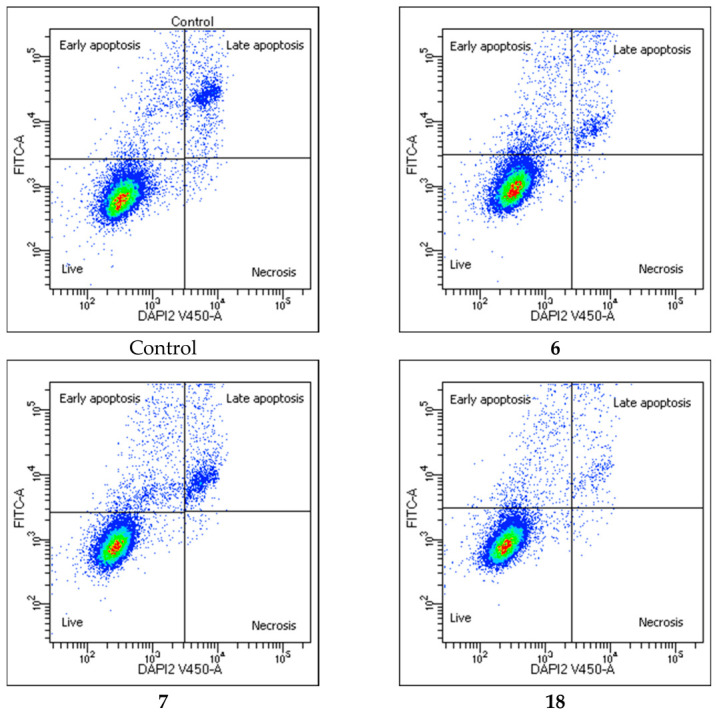
Apoptotic activity of K562 cells treated with pyrrolo[3,4-*d*]isoxazolines **6**, **7**, **18** at a concentration of 10 μg/mL for 24 h.

**Figure 11:**
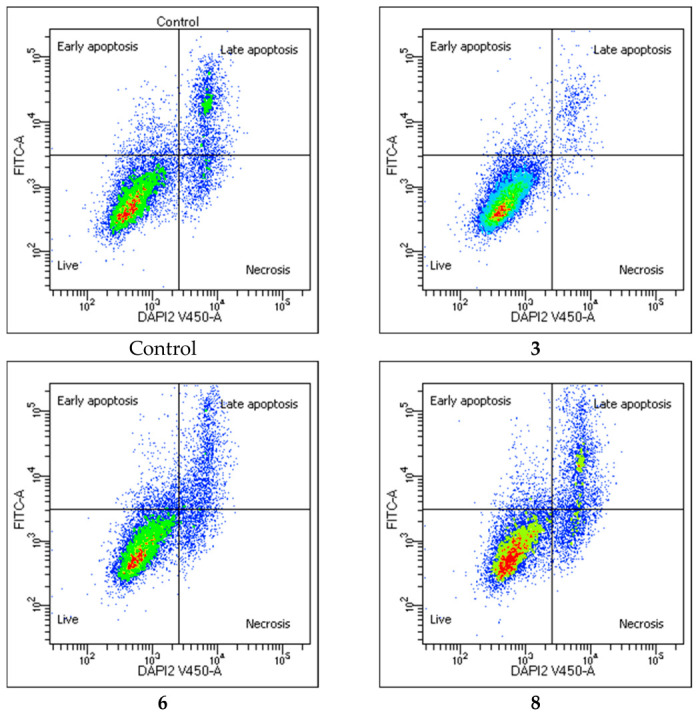
Apoptotic activity of HeLa cells treated with pyrrolo[3,4-*d*]isoxazolines **3**, **6**, **8** at concentration of 10 μg/mL for 24 h.

The percentage of apoptosis induced by pyrrolo[3,4-*d*]isoxazolines **3**, **6**–**9**, **17**, **18** in K562 and HeLa cells was detected by applying Annexin V/ DAPI double-staining flow cytometry [75]. The cells were treated with DMSO as a control. The results showed that the studied pyrrolo[3,4-*d*]isoxazolines did not cause a significant pro-apoptotic effect. Indeed, the proportion of K562 cells in early apoptosis just slightly increased from 5.5 ± 0.2 for control to 10.2 ± 0.8 for isoxazoline 6, while the proportion of cells in late apoptosis decreased from 10.2 ± 0.4 to 5.1 ± 0.3. In total, the proportion of apoptotic cells is in the range 10–17% as compared to 15% for control, which means there is no significant effect, but the absence of necrotic activity should also be noted. A comparable effect was also observed for HeLa cells following exposure to the studied compounds. Certain representative results are shown in Figure 10 and Figure 11 and Table 5.

## 3. Materials and Methods

### 3.1. Chemistry

All the pyrrolo[3,4-*d*]isoxazolines **1**–**9** and pyrrolo[2,1-*a*]isoquinolines **10**–**18** were prepared according to the literature data [47,48,49]. Starting maleimides were prepared as described in [76], and hydroxymoyl chlorides were prepared as described in [77] and [78]. All the compounds were isolated in their racemic form. Their NMR spectra (recorded in CDCl_3_ on a Bruker Avance III spectrometer (400 MHz, Bruker Biospin, Rheinstetten, Germany)) were identical to those described by us earlier [47,48,49]. Purity and individuality of the compounds were monitored by TLC and NMR.

#### 3.1.1. General Procedure for the Synthesis of Bicyclic Isoxazolines **1**–**5**

A solution of 1.2 equiv of triethylamine in 10 mL of dry benzene was slowly added to a vigorously stirred solution of 1.0 equiv of maleimide and 1.2 equiv of hydroxymoxyl chloride in 15 mL of dry benzene. The mixture was allowed to stand at room temperature for 12 h (until completion of the reaction by TLC control) and then decomposed with 10 mL of water. The organic layer was separated, and the aqueous layer was treated with methylene chloride (2 × 5 mL). The organic layers were combined and dried over Na_2_SO_4_. The solvent was evaporated in a vacuum, and the residue was crystallized from diethyl ether.

#### 3.1.2. General Procedure for the Reduction of Bicyclic Isoxazolines with Sodium Borohydride to Hydroxylactams **6**–**9**

In total, 3 equiv of sodium borohydride was slowly added to a solution of 1 equiv of corresponding bicyclic isoxazoline in a mixture of 9 mL methylene chloride and 3 mL ethanol at –78 °C. The reaction mixture was stirred at this temperature for 1 h and then left to stand in a refrigerator at –20 °C until reaction completion (TLC control). When the imide was no longer detected, the mixture was decomposed with saturated aqueous ammonium chloride, extracted with methylene chloride (2 × 5 mL), and dried over Na_2_SO_4_. The solvent was evaporated in a vacuum to obtain hydroxylactams **6**–**9**, which were further used without purification.

#### 3.1.3. General Procedure for the Preparation of Substituted Isoxazolopyrroloisoquinolines **10**–**18** by Cyclization of Hydroxylactams **6**–**9**

A vigorously stirred solution of hydroxylactams (**6**–**9**) in 5 mL of anhydrous dichloromethane was added to 3 equiv of boron trifluoride diethyl etherate under an argon atmosphere. The reaction mixture was stirred at room temperature in a capped vial for 16 h. After completion of the reaction (TLC control), 10 mL of water was added carefully to the reaction mixture. The aqueous layer was extracted with dichloromethane (3 × 5 mL), the organic layers were combined, dried over Na_2_SO_4_ and evaporated to dryness. The residue was then purified by preparative TLC on a silica gel column using a mixture of CH_2_Cl_2_/MeOH as eluent.

#### 3.1.4. In Silico Analysis

The molecular descriptors of the synthesized pyrrolo[3,4-*d*]isoxazolines **1**–**9** and pyrrolo[2,1-*a*]isoquinolines **10**–**18** were determined with the widely used free online software SwissADME ADMETlab 3.0 (http://www.swissadme.ch/ accessed on 25 May 2026) and analyzed according to Veber’s rule and Lipinski’s rule.

ADMET profiling was estimated in silico using the ADMETlab 3.0 online software (https://admetlab3.scbdd.com accessed on 25 May 2026).

### 3.2. Cell Culture and Culturing Conditions

Human embryonic kidney (HEK293T), cervical carcinoma (HeLa), and erythroleukemia (K-562) cell lines were sourced from the Cell Culture Collection of the Institute of Cytology, RAS. The human melanoma (Sk-mel-2) cell line was sourced from the Cell Culture Collection of the Institute of Cytology and Genetics, Siberian Branch of RAS. K-562 cells were maintained in RPMI medium (Hyclone, GE Healthcare Life Sciences, Logan, UT, USA) supplemented with 10% (*v*/*v*) fetal bovine serum (Hyclone) and gentamicin (50 μg/mL, Sigma-Aldrich, St. Louis, MO, USA). HEK293T, HeLa, and Sk-mel-2, were maintained in Dulbecco’s Modified Eagle’s Medium (DMEM, Hyclone) with the same supplements. The cell lines were maintained at 37 °C in a humidified atmosphere containing 5% CO_2_.

### 3.3. Cell Proliferation Assay

Cell viability was assessed in vitro using the MTS assay. Briefly, cells were seeded in 96-well microtiter plates at 5 × 10^3^ cells per well in 100 μL of complete medium and allowed to proliferate and adhere for 24 h at 37 °C. Then, cells were exposed to varying concentrations of test compounds for 1 and 3 days. After the treatment, 20 μL of MTS reagent (BioVision, Milpitas, CA, USA) stock solution was added to each well, and the plates were incubated for 2 h at 37 °C in a humidified 5% CO_2_ atmosphere. Absorbance was measured at 495 nm using a Multiskan GO plate reader (Thermo Fisher Scientific, Waltham, MA, USA). All conditions were run in triplicate.

### 3.4. Actin Cytoskeleton Staining

HeLa and Sk-mel-2 cells were seeded onto a Petri dish with cover slips at a density of 2 × 10^5^ cells per dish and incubated for 24 h. Then, cells were exposed to the selected compounds (10 μg/mL) for 24 h. After treatment, the medium was removed and cells were fixed with 4% paraformaldehyde (Sigma-Aldrich, St. Louis, MO, USA), washed three times with PBS, and permeabilized with 0.3% Triton-X100 (Sigma-Aldrich, St. Louis, MO, USA). Cells were rinsed three times with PBS. Actin filaments were stained with rhodamine-phalloidin (Invitrogen, Carlsbad, CA, USA) for 15 min at 37 °C. Samples were washed three times with PBS and mounted in Fluoroshield medium (Sigma-Aldrich, St. Louis, MO, USA). Staining intensity was evaluated using an AxioObserver Z1 confocal microscope (Carl Zeiss MicroImaging GmbH, Jena, Germany). At least 30 cells were imaged per experiment. Images were processed using ImageJ software 1.54g.

### 3.5. Evaluation of Cell Motility by Scratch Test

Cells were plated in Petri dishes at a density of 5 × 10^5^ cells per dish and cultured until they reached confluence. Scratch wounds were then created using a 200 μL pipette tip and detached cells were removed by washing with PBS. The medium was replaced with serum-free DMEM to suppress cell proliferation, after which the test compounds were added at IC_70_–IC_80_ concentrations and the cultures were incubated for 24 h. Wound areas were examined under bright-field microscopy, photographed at 0 and 24 h, and images were acquired with an Axio Observer Z1 confocal microscope (Carl Zeiss MicroImaging GmbH, Jena, Germany). The percent of wound closure in five randomly chosen fields was calculated with NIH ImageJ software.

### 3.6. Annexin V-FITC/DAPI Staining Assay

Twenty-four-well plates were used to seed the cells (5 × 10^4^ cells per well). Cells were incubated for 24 h, then treated with studied compounds for 24 h. Then, cells were washed, harvested by trypsinization, stained with Annexin V-FITC (BD FACSCanto II, Becton Dickinson, San Jose, CA, USA) and DAPI (Thermo Fisher Scientific, Waltham, MA, USA), in accordance with the manufacturer’s protocol, and analyzed by flow cytometry. The proapoptotic effect of studied compounds was evaluated by an Annexin V-FITC/DAPI (AV/DAPI) dual-staining assay to examine the occurrence of phosphatidylserine externalization, which facilitated the detection of early apoptotic cells (upper-left quadrant; AV+/DAPI–), late apoptotic cells (upper-right quadrant; AV+/DAPI+), live cells (lower-left quadrant; AV–/DAPI–), and necrotic cells (lower-right quadrant; AV–/DAPI+) [79].

### 3.7. Statistical Analysis

Statistical analysis was carried out using Statistica 6.0. Data from three independent experiments were expressed as mean ± standard deviation and compared using Student’s *t*-test.

## 4. Conclusions

We have studied a series of pyrrolo[3,4-*d*]isoxazolines and derived from them pyrrolo[2,1-*a*]isoquinolines as potential antitumor agents. The antiproliferative activity of the products was screened against human erythroleukemia (K562), cervical carcinoma (HeLa), melanoma (Sk-mel-2), and embryonic kidney (HEK293T) cell lines. Most effective among the screened compounds were bicyclic hydroxylactams **6**–**9**; they showed IC_50_ values ranging from 12 to 36 mg/mL for all tested cell lines with selectivity indexes up to 12. Non-reduced bicyclic isoxazolines **1**–**5** and prepared by cyclization of hydroxylactams isoxazolopyrroloisoquinolines **10**–**18** showed, in general, lower activity. We observed by confocal microscopy that treatment of HeLa and Sk-Mel-2 cells with pyrrolo[3,4-*d*]isoxazolines or derived from them pyrrolo[2,1-*a*]isoquinolines induced pronounced alterations in the actin cytoskeleton (loss of stress fibers with diffuse redistribution of granular actin throughout the cytoplasm in up to 25% of treated cells, decrease in filopodia-like protrusions up to 69%). Such cytoskeletal changes and proposed altered cell motility were confirmed by scratch-test (revealed a three-fold decrease in cell motility). No significant pro-apoptotic effect was detected, but the absence of necrotic activity should also be noted. Notably, even compounds with considerably lower proliferative activity still exert a meaningful impact on cytoskeletal structure organization. The experimental findings support the pursuit of pharmacologically active substances within pyrrolo[3,4-*d*]isoxazoline derivatives.

## Figures and Tables

**Table 1:** Physicochemical profiles of compounds according to Veber’s and Lipinski’s rules.

Compound	MW	nHBD	nHBA	Log P	nRotB	TPSA, Å^2^	N_Violation_		Meet Lipinski Criteria	Meet Veber Criteria
							Lipinski	Veber		
	<500	<5	<10	≤5	<10	<140	<1	0	Yes/No	Yes/No
**1**	384.43	0	4	3.39	4	58.97	0	0	Yes	Yes
**2**	404.85	0	4	3.66	4	58.97	0	0	Yes	Yes
**3**	368.81	0	4	3.19	4	58.97	0	0	Yes	Yes
**4**	430.88	0	4	4.03	5	58.97	0	0	Yes	Yes
**5**	410.46	0	4	3.77	5	58.97	0	0	Yes	Yes
**6**	386.44	1	4	3.06	4	62.13	0	0	Yes	Yes
**7**	370.83	1	4	2.86	4	62.13	0	0	Yes	Yes
**8**	412.48	1	4	3.43	5	62.13	0	0	Yes	Yes
**9**	432.90	1	4	3.70	5	62.13	0	0	Yes	Yes
**10**	368.43	0	3	3.48	1	41.90	0	0	Yes	Yes
**11**	388.85	0	3	3.75	1	41.90	0	0	Yes	Yes
**12**	332.40	0	3	3.02	1	41.90	0	0	Yes	Yes
**13**	352.81	0	3	3.29	1	41.90	0	0	Yes	Yes
**14**	394.47	0	3	3.85	2	41.90	0	0	Yes	Yes
**15, 16, 17**	414.88	0	3	4.12	2	41.90	0	0	Yes	Yes
**18**	318.37	0	3	2.80	1	41.90	0	0	Yes	Yes

MW: molecular weight; nHBD: number of hydrogen-bond donors; nHBA: number of hydrogen-bond acceptors; Log P: logarithm of partition coefficient of the compound between n-octanol and water; nRotB: number of rotatable bonds; TPSA: topological polar surface area; N_Violation_: number of violated criteria.

**Table 2:** ADMET profiles of selected compounds.

Compound	HIA	BBB	PPB, %	CYP_2D6 Inhibition	LogS	Carcinogenicity	Rat Oral Acute Toxicity	AMES Toxicity	hERG Blockers
**1**	---	+	98.7	---	−4.904	0.469	0.55	0.702	0.234
**2**	---	+++	98.9	---	−5.092	0.399	0.631	0.561	0.353
**3**	---	+++	98.5	---	−4.612	0.148	0.482	0.252	0.208
**4**	---	+++	99.1	+++	−4.893	0.048	0.814	0.129	0.604
**5**	---	--	98.4	---	−5.203	0.124	0.575	0.33	0.223
**6**	---	++	98.5	---	−4.458	0.198	0.481	0.429	0.252
**7**	---	+++	98.7	---	−4.386	0.047	0.464	0.112	0.241
**8**	---	+++	98.4	---	−4.734	0.054	0.532	0.187	0.213
**9**	---	+++	98.8	+++	−4.558	0.01	0.753	0.054	0.555
**10**	---	+++	98.4	---	−4.933	0.413	0.749	0.645	0.184
**11**	---	+++	98.8	--	−5.213	0.345	0.807	0.497	0.286
**12**	---	+++	97.8	---	−4.269	0.229	0.628	0.296	0.075
**13**	---	+++	97.8	---	−4.269	0.182	0.628	0.296	0.075
**14**	---	+++	98.5	---	−4.937	0.163	0.631	0.363	0.144
**15**	---	+++	98.8	---	−5.154	0.128	0.706	0.237	0.23
**16, 17**	---	+++	98.6	+++	−5.045	0.119	0.736	0.233	0.363
**18**	---	+++	97.9	---	−4.033	0.314	0.614	0.471	0.098

HIA: human intestinal absorption; BBB: in vivo blood–brain barrier penetration (C. brain/C. blood); PPB: in vitro plasma protein binding; CYP_2D6_inhibition: in vitro Cytochrome P450 2D6 inhibition; logS: logarithm of aqueous solubility value; rat oral acute toxicity: carcinogenicity bioassay in rat; hERG blockers: in vitro human ether-a-go-go related gene channel inhibition. Value of HIA, BBB, CYP2D6 Inhibition: 0–0.1 (---), 0.1–0.3 (--), 0.5–0.7 (+), 0.7–0.9 (++), 0.9–1.0 (+++).

**Table 3:** IC_50_ values of studied derivatives against K562, HeLa and Sk-mel-2 cell lines for 72 h.

Compound	IC_50_, μg/mL			Compound	IC_50_, μg/mL		
	K562	HeLa	Sk-mel-2		K562	HeLa	Sk-mel-2
**1**	18 ± 1	41 ± 3	>50	**11**	25 ± 2	41 ± 3	39 ± 2
**2**	>50	>50	43 ± 2	**13**	>50	47 ± 4	>50
**3**	35 ± 2	37 ± 2	25 ± 2	**15**	33 ± 2	>50	42 ± 3
**6**	19 ± 2	13 ± 4	13 ± 4	**17**	23 ± 2	>50	12 ± 2
**7**	16 ± 1	20 ± 1	27 ± 2	**18**	16 ± 2	>50	12 ± 4
**8**	24 ± 2	15 ± 2	>50	Doxorubicin	1 ± 0.3	7 ± 2	6 ± 1
**9**	24 ± 2	12 ± 1	36 ± 2				

**Table 4:** SI values of most active derivatives for 72 h.

Compound	IC_50_, μg/mL						
	HEK293T	K562	SI	HeLa	SI	Sk-mel-2	SI
**3**	107 ± 10	35 ± 2	3.1	37 ± 2	2.9	25 ± 2	4.3
**6**	22 ± 4	19 ± 2	1.2	13 ± 4	1.7	13 ± 4	1.7
**7**	100 ± 10	16 ± 1	6.2	20 ± 1	5.0	27 ± 2	3.7
**8**	92 ± 8	24 ± 2	3.8	15 ± 2	6.1	>50	<1.8
**9**	144 ± 14	24 ± 2	6.0	12 ± 1	12.0	36 ± 2	4.0
**17**	23 ± 4	23 ± 2	1.0	>50	<1	12 ± 2	1.9
**18**	38 ± 6	16 ± 2	2.4	>50	<1	12 ± 4	3.2

SI: selectivity indexes.

**Table 5:** Apoptotic activity of K562 and HeLa cells treated with pyrrolo[3,4-*d*]isoxazolines **3**, **6**–**9** at concentration of 10 μg/mL for 24 h.

Cell Line	Compound	Live Cells, %	Early Apoptotic Cells, %	Late Apoptotic Cells, %	Necrotic Cells, %
K562	Control	83.6 ± 0.6	5.5 ± 0.2	10.2 ± 0.4	0.7 ± 0.1
	**3**	83.3 ± 0.3	7.9 ± 0.2	8.2 ± 0.2	0.5 ± 0.1
	**6**	84.5 ± 0.8	10.2 ± 0.8	5.1 ± 0.3	0.2 ± 0.2
	**7**	82.5 ± 0.8	7.8 ± 0.4	9.4 ± 0.6	0.3 ± 0.1
	**8**	83.6 ± 1.1	8.5 ± 1.0	7.5 ± 0.4	0.4 ± 0.2
	**9**	88.0 ± 0.5	6.9 ± 0.4	4.9 ± 0.2	0.2 ±0.1
	**17**	86.8 ± 0.8	8.3 ± 0.5	4.7 ± 0.4	0.3 ± 0.1
	**18**	89.5 ± 0.2	7.3 ± 0.2	3.0 ± 0.2	0.2 ± 0.1
	DMSO	13.1 ± 0.5	23.8 ± 0.6	62.9 ± 0.2	0.1 ± 0.1
HeLa	Control	68.9 ± 2.0	7.1 ± 2.6	16.8 ± 1.4	7.2 ± 1.9
	**3**	91.0 ± 1.7	4.3 ± 1.6	4.0 ± 0.4	0.6 ± 0.3
	**6**	64.8 ± 1.3	14.7 ± 3.2	16.5 ± 0.5	4.0 ± 1.5
	**7**	76.5 ± 5.5	9.8 ± 3.8	11.0 ± 2.7	2.7 ± 1.0
	**8**	62.1 ± 2.2	8.0 ± 1.1	21.3 ± 3.0	8.7 ± 1.4
	**9**	75.4 ± 2.4	9.9 ± 3.3	10.8 ± 1.1	3.9 ± 1.5
	DMSO	31.5 ± 2.7	15.7 ± 4.3	51.6 ± 0.7	1.2 ± 1.0

## Data Availability

The data presented in this study are available on reasonable request from the corresponding authors.
